# Anomalous systemic arterial supply to normal lung

**DOI:** 10.36416/1806-3756/e20240034

**Published:** 2024-05-08

**Authors:** Edson Marchiori, Bruno Hochhegger, Gláucia Zanetti

**Affiliations:** 1. Universidade Federal do Rio de Janeiro, Rio de Janeiro (RJ) Brasil.; 2. University of Florida, Gainesville (FL) USA.

A 47-year-old woman presented with acute respiratory infection. She underwent a chest CT that showed an anomalous vessel originating from the aorta and supplying the left lower lobe (Figure 1). Venous drainage and the regional bronchial tree were normal. The final diagnosis was anomalous systemic arterial supply to normal lung.

**Figure 1:**
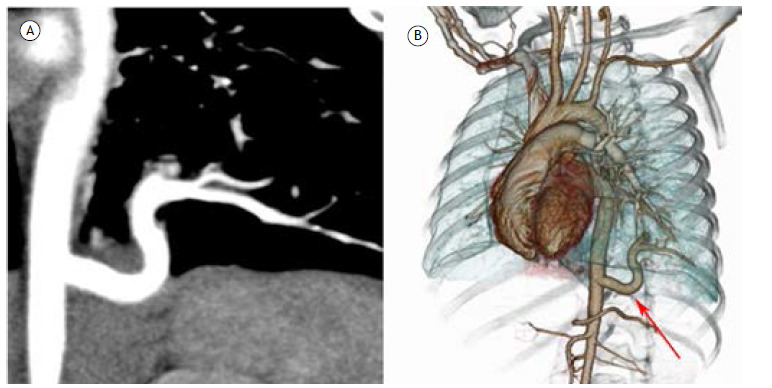
Anomalous systemic arterial supply to normal lung. In A, CT angiography shows an anomalous vessel originating directly from the aorta and heading to the left lower lobe. In B, three-dimensional reconstruction shows the anomalous artery (red arrow) and normal venous drainage of the left lung. In addition, the regional bronchial tree (not shown) was normal.

Systemic arterial supply to normal basal segments of the lung with no sequestration is a rare congenital anomaly, characterized by the presence of an anomalous systemic artery feeding an area of normal lung parenchyma. This change was previously classified as a subtype of pulmonary sequestration. However, it differs from classic bronchopulmonary sequestration because the involved lung tissue maintains a normal connection with the bronchial tree. Furthermore, the absence of the interlobar artery distal to the superior segmental artery is an important differential from the classic bronchopulmonary sequestration. Venous return occurs through the inferior pulmonary vein to the left atrium. There is no direct communication between the anomalous arterial vessels and the veins of the basal segments. The basal segments of the left lower lobe are most commonly involved. Clinical manifestations of this anomaly are diverse. The majority of adult patients are asymptomatic, and the alteration is incidentally found in imaging tests performed for other purposes. Symptomatic adults generally present with hemoptysis or dyspnea, whereas the most common clinical manifestation in pediatric patients is heart murmur.^1^ ^,^ ^2^

The examinations of choice for diagnosis is contrast-enhanced CT that shows the anomalous artery, which generally originates from the descending aorta, but it can also arise from the proximal abdominal aorta or the celiac trunk, heading toward the basal segments of the lower lobes, especially the left one. CT can also provide information about the normal morphology of the bronchial tree and lung parenchyma. Venous drainage is also normal. Another finding is the absence of the interlobar artery distal to the superior segmental artery. These tomographic findings make it possible to differentiate it from the classic bronchopulmonary sequestration. Treatment depends on the age at diagnosis and the existence of other associated pulmonary anomalies. In adults, the anomalous connection is usually treated with lobectomy or surgical ligation of the anomalous artery. Surgery is indicated, because the condition presents potential risks, such as hemoptysis due to pulmonary hypertension, heart failure due to left-to-left shunt, and infection. In the majority of reported cases, lobectomy or segmentectomy was performed. Our patient, because she was asymptomatic, refused surgery and was advised to undergo annual periodic control.^1^ ^,^ ^2^

## References

[1] Singhi AK, Nicholson I, Francis E, Kumar RK, Hawker R (2011). Anomalous systemic arterial supply to normal basal segment of the left lung. Heart Lung Circ.

[2] Do KH, Goo JM, Im JG, Kim KW, Chung JW, Park JH (2001). Systemic arterial supply to the lungs in adults spiral CT findings. Radiographics.

